# Pre-pandemic sleep behavior and adolescents’ stress during Covid-19: a prospective longitudinal study

**DOI:** 10.1186/s13034-021-00399-x

**Published:** 2021-08-30

**Authors:** Reut Gruber, Gabrielle Gauthier-Gagne, Denise Voutou, Gail Somerville, Sujata Saha, Johanne Boursier

**Affiliations:** 1 Attention, Behaviour and Sleep Lab, Douglas Mental Health University Institute, 6875 LaSalle Blvd, Montreal, QC H4H 1R3 Canada; 2 Department of Psychiatry, Faculty of Medicine, McGill University, Montreal, QC Canada; 3 Faculty of Medicine, Integrated Program in Neuroscience, McGill University, Montreal, QC Canada; 4 Riverside School Board, Saint-Hubert, QC J3Y 0N7 Canada

**Keywords:** Sleep, Adolescents, Stress, COVID-19, Prospective, Longitudinal

## Abstract

**Objective:**

To prospectively document changes in adolescents’ sleep before versus during the COVID-19 pandemic, and to examine their impact on adolescents’ perceived stress.

**Methods:**

Sixty-two typically developing adolescents participated in the study before (Time 1: January 15 to March 13, 2020) and during (Time 2: May 15 to June 30, 2020) the COVID-19 pandemic in Canada. At Time 1, each participant’s sleep pattern was assessed in the home environment using actigraphy and sleep logs for seven consecutive nights. Adolescents completed a battery of questionnaires in which they reported on their sleep schedule, duration, and quality, as well as their activities at bedtime, their daytime sleepiness, and their social/emotional behavior. The participants’ parents provided demographic information. At Time 2, each participant completed a sleep log, the same battery of questionnaires regarding sleep, and the Perceived Stress Scale.

**Results:**

(1) Adolescents’ reported sleep was of longer duration and on a delayed schedule during the COVID-19 pandemic compared to pre-pandemic. (2) A larger proportion of adolescents reported meeting or exceeding the recommended amount of sleep during the COVID-19 pandemic compared to pre-pandemic sleep. (3) “Social jet lag” disappeared during the COVID-19 pandemic. (4) A shorter reported sleep duration and higher level of arousal at bedtime at Time 1 were significant predictors of adolescents’ perceived stress at Time 2—during the COVID-19 pandemic. (5) A higher levels of arousal at bedtime and lower reported sleep quality at Time 2 were concurrently associated with higher levels of perceived stress among adolescents, even when we controlled for the levels of pre-pandemic emotional or behavioral issues, sleep duration, or sleep quality.

**Conclusion:**

Sleep duration and cognitive-emotional arousal, which are both modifiable behaviors, were associated with adolescents’ perceived stress during the COVID-19 pandemic. These behaviors could be useful targets for preventive interventions aiming to reduce adolescents’ stress in the face of stressogenic situations, such as the COVID-19 pandemic.

## Introduction

The novel coronavirus disease, COVID-19, emerged in Wuhan, China, in December 2019. 1 By January 27, 2020, the virus was confirmed to have reached Canada. The first case of community transmission in Canada was confirmed on March 5, and province-wide school closures and stay-at-home orders were announced in many provinces by March 13, 2020.

The pandemic lockdown interrupted the continuity of learning and triggered social isolation, changes in routine, cancelation of out‐of‐home leisure time activities, loss of the sense of security and safety, and fears about the future [1]. A recent analysis projected that serious mental health problems caused by the pandemic will outlast its physical impact and will still be seen by 2029, suggesting that today’s elevated mental health challenges will continue well beyond the coronavirus outbreak itself [2].

The COVID-19 pandemic increased levels of uncertainty and psychological stress in adolescents around the world [3–16]. Perceived stress, which refers to the degree to which a person assesses events in their life as being stressful, unpredictable, and uncontrollable [17, 18] is a psychological risk factor that contributes to the onset and development psychiatric symptoms among adolescents [19–22].

Adolescence is a critical developmental period [23]. Half of all mental health disorders emerge or worsen during this period and the consequences of these disorders “stick” long into adulthood [24, 25]. It is therefore essential that we identify modifiable, age-appropriate targets for interventions that can lower adolescents’ perceived stress.

Inadequate sleep is a significant risk factor for increased stress levels [26–30], whereas optimized sleep is linked to reduced stress [29, 31]. Identifying specific sleep behaviors that can contribute to or reduce high level of stress would be important because such work could allow us to identify modifiable targets for interventions aimed at lowering adolescents’ stress and reducing the pathogenic impact of stress on their mental health.

Studies conducted to examine adolescents’ sleep during the COVID-19 pandemic have found that, among typically developing adolescents, sleep schedules were delayed [32–34], sleep duration was extended, and daytime sleepiness decreased [34–36]. Results regarding the prevalence or severity of sleep disruptions have been inconsistent, with some studies reporting that disruptions worsened [32, 37–39] while others found that they improved [40].

This rapidly evolving body of knowledge regarding adolescents’ sleep during the COVID-19 pandemic offers important information. However, most of the studies have weaknesses that have limited their contributions. With three exceptions, all of the studies were either cross-sectional [36, 39] which does not allow causal inferences to be made regarding the impact of COVID-19 on sleep, or retrospective [38, 41] and therefore, subjected to recall bias. The three exceptions were longitudinal studies. Of them, one was qualitative [35]. The other longitudinal studies [32, 34] used baseline data that were collected a full year [32] before the pandemic or after the COVID-19 lockdown had already been lifted for a portion of the sample [34]; these data thus could not have accurately captured changes in sleep as they happened following the pandemic, nor could they rule out the potential impact of season or maturation on the participants’ daily routines and therefore their sleep patterns. In addition, participants in these studies were not subjected to the same changes in their school schedules or routines, meaning that the pandemic could have had inconsistent effects on their sleep patterns.

Another weakness is that most of the previous studies recruited participants via online surveys e.g. [42] most of which were anonymous e.g. [38]. It was therefore impossible to confirm the identity of participants or determine the validity of their reported diagnoses or health status. Finally, the previous studies all failed to: control for the potential impact of pre-existing physical, emotional, or behavioral problems that could have affected adolescents’ pre-pandemic sleep or their response to the pandemic; measure behaviors at bedtime known to affect sleep; or prospectively examine adolescents’ stress as it relates to their sleep.

The present study sought to fill these gaps by: (1) including carefully characterized participants; (2) using a prospective longitudinal design; (3) enrolling participants who were exposed to similar school schedules before and during the COVID-19 pandemic and were exposed to similar societal changes following the pandemic, thereby avoiding potential bias regarding how the school schedules might have affected sleep or sleep-related behaviors; and (4) prospectively measuring adolescents’ stress using a valid measurement scale. Moreover, our study utilized particularly suitable time points. Time 1 spanned from January 15 to March 12, 2020. Due to the COVID-19 pandemic, Canadian schools closed on March 13, 2020. Time 2 spanned from April 28 to June 3, 2020. During this time, the schools had re-opened and classes were being taught in a remote-teaching format (for details see [35]). These time points were ideally suited for prospectively evaluating the immediate impact of COVID-19 on adolescents’ sleep behavior, especially since since the sampling times enabled us to capture sleep changes observed following the pandemic. Time 2 occurred 6 weeks after Time 1, and the studied period included the point at which school closures took place. The brief gap between Time 1 and Time 2 allowed the participants to make an initial adjustment to their new reality but minimized confounding effects of changes in sleep due to seasonal changes or physical maturation.

The objectives of the study were: (1) to describe and compare adolescents’ sleep patterns and behaviors immediately before and during the COVID-19 pandemic; (2) to examine the contributions of multiple sleep dimensions (quality, duration, sleepiness, timing) and behaviors (behaviors/cognitions associated with high levels of arousal) to adolescents’ perceived levels of stress during the COVID-19 pandemic.

We hypothesized that: (1) a short sleep duration, higher daytime sleepiness, and higher arousal at bedtime prior to the COVID-19 pandemic would predict higher levels of perceived stress among adolescents during the COVID-19 pandemic; and (2) a short sleep duration, higher daytime sleepiness, and higher arousal at bedtime during the COVID-19 pandemic would be associated with higher levels of perceived stress among adolescents during the COVID-19 pandemic above and beyond these variables at Time 1.

## Methods

### Participants

Sixty-eight typically developing adolescents between 12–16 years participated at Time 1 (26% male; M = 13.38, SD = 1.3 years) and 62 participated at Time 1 and 2. There was no significant demographic or behavioral difference between the adolescents that participated at Time 2 versus those that did not.

### Procedure

Teachers invited students and parents to participate in the study via flyers. Parents who responded to the flyers were contacted for further screening. Adolescents and parents provided consent and assent, respectively, for data collection at both time points. Prior to a child's enrollment, their eligibility was determined by screening for the absence of sleep disorders, significant health problems, and behavioral problems, based on the parents’ responses to a detailed questionnaire regarding the health of their child. The study was approved by the Research Ethics Board of the Douglas Douglas Mental Health University Institute.

At Time 1, each participant’s sleep pattern was assessed in the home environment using actigraphy (AW-64 series; Mini-Mitter, Sunriver, OR, USA) for seven consecutive nights. In addition, each participant completed a daily sleep log and provided information regarding his or her sleep duration and quality, activities at bedtime, and daytime sleepiness. Parents were asked to provide demographic information. At Time 2, data collection was conducted using an online remote survey that included all of the questionnaires used at Time 1, as well as adolescents’ perceived stress. At Time 2, all research involving in-person interaction with human subjects had to be discontinued or postponed due to COVID-19. We did not have an infrastructure that would allow us to conduct contact-free actigraphy data collection, and therefore were not able to conduct actigraphy during this part of the study.

### Measures

#### Sleep

##### Actigraphy (Time 1)

Nighttime sleep was monitored by actigraphy, which has been shown to be a reliable method for evaluating sleep. The utilized Actiware Sleep 6.1 software package (Mini-Mitter) applies a sleep-scoring algorithm that was previously validated and found to display a high degree of correspondence with polysomnographic data [43–46]. The actigraphic data were analyzed in 1-min epochs. The total number of activity events was computed for each epoch; if the threshold sensitivity value of the mean score during the active period was exceeded, the epoch was considered to be waking in nature. Otherwise, the epoch was considered to be sleep. The studied sleep parameters included: (1) the sleep schedule, including the times of sleep start and sleep end; (2) the sleep duration, which was defined as the sum of epochs between sleep onset and sleep end that were scored as “sleep” according to the algorithm; and (3) the sleep efficiency, which was defined as the percentage of time in bed spent sleeping. These measures were averaged separately over the 5 weeknights and 2 weekend nights, allowing us to examine the childrens’ habitual sleep patterns immediately prior to the COVID-19 pandemic.

##### Sleep log (Time 1, Time 2)

The participating adolescents were asked to record their daily bedtimes and wake times.

##### Self-reported sleep duration (Time1, Time 2)

The participating adolescents were asked to indicate how many hours of actual nightly sleep they obtained during the past month.

##### Self-reported sleep quality (Time 1, Time 2)

The participating adolescents were asked to rate their sleep quality during the past month on a 0–3 scale, with higher scores indicating worse quality. The question was stated as: “During the past month, how would you rate your sleep quality overall? 0 very good; 1 fairly good; 2 fairly bad; 3 very bad”.

The Modified Epworth Sleepiness Scale: (Time 1, Time 2) was used to record daytime sleepiness [47]. The scale consists of eight items examining the propensity for a child to fall asleep in various everyday situations; scores range from 0 = no chance to 3 = a high chance of dozing for each of the eight items, for a maximum score of 24. Higher scores indicate a higher level of sleepiness. The Modified Epworth Sleepiness Scale is a reliable (test–retest reliability 0.89) and internally valid scale for adolescents aged 12–18 years [48].

Arousal at bedtime: (Time 1, Time 2) was measured using the behavioral and cognitive-emotional arousal subscales of the Adolescent Sleep Hygiene Scale [49] (internal consistency: α = 0.60 and α = 0.81, respectively) [50]. These sub-scales measure how often behaviors associated with high levels of arousal occurred around bedtime, with each item rated on a 6-point scale (from 1 [never, 0%] to 6 [always, 100%]). Scores are reversed, so higher scores indicate better sleep hygiene, and each subscale score is the mean of all its items.

#### Dependent (outcome) measure

The Perceived Stress Scale [51] and its derivatives are among the most commonly used self-report measures of subjective global stress. The PSS-10 measures the degree to which one perceives aspects of one’s life as being uncontrollable, unpredictable, and overloading. Participants are asked to respond to each question on a 5-point Likert scale ranging from 0 (never) to 4 (very often), indicating how often they have felt or thought a certain way within the past month. Scores range from 0 to 40, with higher scores indicative of greater perceived stress. The PSS-10 has been validated among adolescents [17, 52, 53] including adequate internal reliability, and acceptable internal reliability in this study (Cronbach’s α = 0.83) [51].

### Control variables

Behavioral/emotional problems (Time 1) were assessed using the Youth Self Report (YSR) [54] a 112-item self-report designed for children and adolescents (ages 11–17) that assesses behavioral competency and problems. The adolescent selected his or her response from 0 (not true) to 2 (very true or often true), and overall behavioral and emotional functioning were measured by the total problem scale. Achenbach reported the mean 7-day test–retest reliability for the problem scales was 0.65 for 11 to 14-year-old adolescents and 0.83 for 15 to 18-year-old adolescents [54].

Demographic information: (Time 1) regarding the caregivers’ education, marital status, and household income was collected through a background questionnaire.

Health information: (Time 1, Time 2) and information regarding changes in caregivers’ occupational or health status during the COVID-19 pandemic (Time 2) were collected using a detailed parental questionnaire.

### Analysis

All statistical analyses were performed using SPSS 25 (www.spss.com). An a priori α was set at *P* < 0.05 for all statistical analyses.

Descriptive statistics were computed to examine the demographic characteristics of the sample. A linear interpolation method was applied to infer missing values. The demographic and clinical features did not differ between youth with complete (N = 51) versus partially imputed (N = 10) data [55].

Descriptive analysis was used to describe actigraphy-measured sleep parameters obtained before COVID-19 (Time 1) and using self-reported sleep parameters during COVID-19 (Time 2). Paired-samples t-tests were conducted to compare participants’ weekday vs. weekend bedtimes and wake up times (“social jet lag”) at each time point. To assess participants’ ability to accurately judge their sleep patterns, Pearson product-moment correlation analysis was used to measure the associations between the actigraphic data and self-reported bedtime, wake up time, sleep duration, and sleep quality at Time 1.

To assess changes in sleep patterns and behavior following initiation of the COVID-19 pandemic, we reclassified participants’ self-reported sleep durations into three categories: < 8 h, 8–10 h, and > 10 h [56]. We used the Wilcoxon signed-rank test with time (pre-/during COVID-19) as a repeating variable to compare the proportion of adolescents who obtained sleep durations below, within, or above the amount of sleep recommended for adolescents (8–10 h). We then compared the adolescents’ sleep duration, quality, sleepiness, and arousal at bedtime at Time 1 versus Time 2 using repeated measures multivariate analysis of variance (MANOVA) with time (Time 1, pre-COVID19; Time 2, during COVID19) as the within-subject factor. When the MANOVA yielded a main effect for time, follow-up univariate tests were conducted.

Before proceeding with regression analysis, we examined the potential confounding influence of participants’ characteristics and demographics on the main study outcomes (adolescents’ perceived stress during the COVID-19 pandemic). Consistent with recommendations [57, 58], variables that were significantly associated with the dependent variable were retained as covariates in subsequent analyses.

Hierarchical regression analysis was conducted to: (1) examine the predictive role of pre-COVID19 (Time 1) sleep patterns, sleep behavior, and daytime sleepiness for adolescents’ perceived stress during the COVID-19 pandemic above and beyond demographics and confounders; and (2) determine the cross-sectional associations between sleep patterns, sleep behavior, and daytime sleepiness with adolescents’ perceived stress during the COVID-19 pandemic, above and beyond the expected temporal stability in adolescents’ sleep behavior.

Variables were entered in a pre-determined order: In Step 1, the YSR of behavioral problems pre-COVID-19 and the participant’s gender were entered as control variables. In step 2, pre-COVID 19 self-reported sleep quality, sleep quantity, daytime sleepiness, and cognitive and behavioral arousal at bedtime were entered into the equation. In Step 3, the Time 2 self-reported sleep quality, sleep quantity, daytime sleepiness, and cognitive and behavioral arousal at bedtime were entered into the equation.

The obtained variance inflation factor (all less than 2.0) and collinearity tolerance (all greater than 0.76) suggest that the estimated *β*s were well established in the regression model.

## Results

### Descriptive statistics

Descriptive statistics and demographics are presented in Tables 1, 2, 3, 4.

**Table 1:** Demographics and descriptive statistics

	%	
Household income per year		
$10,000–$25,000	3.6	
$25,000–$45,000	9.1	
$45,000–$65,000	16.4	
$65,000–$95,000	70.9	
$95,000+		
Ethnicity		
Caucasian	87.7	
Asian	3.5	
Multiethnic	5.3	
Other	3.5	
Mother’s marital status		
Married	71.4	
Separated	8.9	
Divorced	5.4	
Remarried	1.8	
Widowed	1.8	
Single	10.7	
Mother’s highest level of education obtained		
High school (nf)	5.4	
High school (f)	12.5	
College/CEGEP (nf)	7.1	
College/CEGEP (f)	17.9	
Undergraduate (nf)	8.9	
Undergraduate (f)	14.3	
Graduate	33.9	
Father’s highest level of education obtained		
High school (nf)	5.6	
High school (f)	16.7	
College/CEGEP (nf)	5.6	
College/CEGEP (f)	25.9	
Undergraduate (nf)	1.9	
Undergraduate (f)	16.7	
Graduate	27.8	
Behavioral/emotional characteristics		
YSR total score		
Girl	53.60	8.66
Boy	52.00	9.92
Total	53.22	8.92

**Table 2:** Objective sleep characteristics measured by actigraphy (Time 1)

	Week night				Weekend night			
	Mean	SD	95% Confidence interval		Mean	SD	95% Confidence interval	
			Lower bound	Upper bound			Lower bound	Upper bound
Bed time								
Girl	22:43	1:10	22:23	23:03	23:24	1:14	23:04	23:46
Boy	23:05	1:18	22:24	23:45	23:38	1:16	22:46	24:05
Total	0.95	1:12	22:31	23:06	23:25	1:14	23:07	23:43
Wake up time								
Girl	7:16	0:47	7:03	7:31	8:16	1:02	7:59	8:34
Boy	7:19	0:43	6:54	7:39	8:12	0:52	7:46	8:39
Total	7:17	0:46	7:05	7:28	8:15	1:00	8:01	8:30
Sleep duration								
Girl	513.90	50.06	499.67	528.13	527.56	71.38	507.48	547.63
Boy	491.80	68.99	456.33	527.27	526.95	61.00	495.59	558.31
Total	508.29	55.75	494.69	521.89	527.40	68.49	510.83	543.98
Sleep efficiency								
Girl	84.41	3.68	83.36	85.45	84.66	4.49	83.40	85.92
Boy	84.09	5.65	81.18	86.99	82.88	6.64	79.46	86.29
Total	84.33	4.22	83.30	85.36	84.22	5.12	82.98	85.45

**Table 3:** Self reported sleep schedule (Time1, Time 2)

	Time 1								Time 2							
	Week Night				Weekend Night				Week Night				Weekend Night			
	Mean	SD	95% Confidence Interval		Mean	SD	95% Confidence Interval		Mean	SD	95% Confidence Interval		Mean	SD	95% Confidence Interval	
			Lower Bound	Upper Bound			Lower Bound	Upper Bound			Lower Bound	Upper Bound			Lower Bound	Upper Bound
Bed time																
Girl	22:28	1:04	22:10	22:46	23:19	1:10	22:59	23:39	24:01	1:40	23:33	24:29	24:06	1:16	23:45	24:28
Boy	22:42	1:16	22:03	23:22	23:12	1:22	22:30	23:55	24:13	2:00	23:11	25:15	24:24	2:17	23:13	25:35
Total	22:31	1:07	22:15	22:48	23:17	1:13	23:00	23:35	24:04	1:44	23:39	24:30	24:11	1:34	23:48	24:33
Wake up time																
Girl	7:25	0:50	7:11	7:40	8:04	1:00	7:47	8:21	9:15	1:25	8:51	9:39	9:18	1:17	8:56	9:40
Boy	7:22	0:48	6:57	7:47	8:01	0:47	7:37	8:25	9:31	1:55	8:32	10:30	9:38	1:22	8:55	10:21
Total	7:25	0:49	7:12	7:37	8:03	0:57	7:49	8:17	9:19	1:33	8:57	9:42	9:23	1:18	9:04	9:42

N = 63

**Table 4:** Descriptive information regarding predictors and outcomes before COVID-19 (Time 1) and during COVID-19 (Time 2)

	Time 1				Time 2			
	Mean	SD	95% Confidence Interval		Mean	SD	95% Confidence Interval	
			Lower Bound	Upper Bound			Lower Bound	Upper Bound
Predictors								
Sleep duration								
Girl	9.12	1.32	8.74	9.50	10.00	1.58	9.56	10.45
Boy	9.10	1.79	8.18	10.02	10.13	0.93	9.65	10.61
Total	9.12	1.44	8.76	9.47	10.03	1.44	9.69	10.38
Sleep quality								
Girl	1.25	0.79	1.02	1.48	0.99	0.77	0.78	1.21
Boy	1.00	0.63	0.66	1.34	0.93	0.56	0.65	1.22
Total	1.19	0.75	1.00	1.38	0.98	0.72	0.80	1.15
Daytime sleepiness (MPESS)								
Girl	7.84	3.56	6.84	8.84	5.20	2.59	4.47	5.93
Boy	6.76	4.05	4.68	8.85	5.28	3.83	3.31	7.25
Total	7.57	3.69	6.68	8.47	5.22	2.91	4.52	5.93
Behavioral arousal								
Girl	2.86	1.14	2.54	3.18	3.06	1.25	2.70	3.41
Boy	2.92	1.30	2.25	3.59	3.43	1.17	2.83	4.03
Total	2.88	1.17	2.59	3.16	3.15	1.24	2.85	3.45
Cognitive emotional arousal								
Girl	3.69	1.01	3.41	3.98	4.27	0.88	4.03	4.52
Boy	4.65	0.68	4.30	4.99	4.60	0.73	4.23	4.98
Total	3.93	1.02	3.69	4.18	4.36	0.85	4.15	4.56
Outcome								
Perceived stress								
Girl	NA				18.45	6.20	16.70	20.19
Boy	NA				15.54	5.53	12.70	18.38
Total	NA				17.72	6.13	16.24	19.21

N = 63

### Sleep patterns

#### Time 1, actigraphy

The actigraphic data collected at Time 1 showed that during the week, participants fell asleep on average at 22:48 and woke up at 7:17. On weekend nights, they fell asleep on average at 23:25 and woke up at 8:15. The differences between week and weekend bedtimes and wake-up times (“social jet lag”) were statistically significant (*t*(67) = − 3.86, *p* < 0.001 and *t*(68) = − 6.89, *p* < 0.001, respectively).

#### Time 1, self-report

The self-reported data collected at Time 1 showed that during the week participants fell asleep on average at 22:31 and woke up at 7:25. On weekend nights, they fell asleep on average at 23:17 and woke up at 8:03. The differences between week and weekend bedtimes and wake up times at Time 1 were statistically significant (*t*(68) = − 5.7, *p* < 0.001 and *t*(46) = − 5.39, *p* < 0.001, respectively).

#### Associations between objective and subjective sleep measures at Time 1

We observed strong correlations between the actigraphy-based and self-reported bedtimes during the week and weekend (*r* = 0.87, *p* < 0.001 and *r* = 0.96, *p* < 0.001, respectively) and wake-up times during the week and weekend (*r* = 0.95, *p* < 0.001 and *r* = 0.72, *p* < 0.005, respectively) in this sample, confirming that the self-reported sleep schedule and duration were a valid method of measuring sleep in this sample.

#### Time 2, self-report

The self-reported data collected at Time 2 revealed that during the COVID-19 pandemic, participants fell asleep on average at 24:04 and woke up at 9:19 during the week, and fell asleep at 24:11 and woke up at 9:23 during the weekend. The differences between week to weekend bedtimes or wake-up times (“social jet lag”) were not statistically significant.

### Percentage of children obtaining the recommended amount of sleep

Table 5 presents the percentages of adolescents who obtained less than, more than, or an amount within the recommended hours of sleep [56] before and during the COVID-19 pandemic. A Wilcoxon signed-rank test indicated that there was a significant difference in the proportion of adolescents obtaining more than the recommended amount of sleep between the two periods (*Z* = − 2.75, *p* < 0.006, *d* = 0.55). As indicated in Table 4, the percentage of adolescents obtaining more than the recommended school-night sleep duration (< 8–10 h) shifted from 4% before COVID-19 to 26.5% during COVID-19.

**Table 5:** Adolescents obtaining recommended sleep duration before COVID-19 (Time 1) and during COVID-19 (Time 2)

Duration before (Time 1) and during (Time 2) COVID-19			Wilcoxon signed-rank test
	Time 1	Time 2	
Self reported sleep duration	%	%	(− 3.26)**
< 8 h	20.0	9.5	
8–10 h	50.9	35.7	
> 10 h	29.1	54.8	

For Wilcoxon Signed-Rank Test tests for sleep duration, variables used included below (< 10), within (8 < duration < 10) and above (> 10) recommended sleep duration < 8 h or ≥ 10 h)

N = 63

^**^p < .01

### Changes in adolescents’ sleep patterns following the initiation of the COVID-19 pandemic

MANOVA conducted to examine changes in adolescents’ sleep following the initiation of the COVID-19 pandemic revealed a significant main effect of time (*F*(7,55) = 17.84, *p* < 0.000, η_p_^2^ = 0.69). Post-hoc univariate analyses revealed that the adolescents’ sleep schedule was significantly delayed at Time 2: Their average bedtime was 1:28 h later and their average wake-up time was 2:13 h later than their pre-pandemic schedules (*F*(1,61) = 68.55, *p* < 0.000, ηp^2^ = 0.53 and *F*(1,61) = 94.33, *p* < 0.000, ηp2 = 0.61, respectively). The adolescents’ sleep duration was 1:01 h longer during the pandemic compared to the pre-pandemic sleep duration (*F*(1,47) = 14.68, *p* < 0.000, ηp^2^ = 0.19). The adolescents’ reported daytime sleepiness and cognitive- emotional arousal at bedtime and behavioral arousal at bedtime were all lower during the pandemic compared to the levels seen pre-pandemic (*F*(1,61) = 13.17, *p* < 0.01, ηp^2^ = 0.18; *F*(1,61) = 4.44, *p* < 0.001, ηp^2^ = 0.22; and *F*(1,61) = 4.2, *p* < 0.05, ηp^2^ = 0.6, respectively). The adolescents’ sleep quality did not change between the two time points.

### Adolescents’ sleep and perceived stress during the COVID-19 pandemic

#### Hierarchical multiple linear regression

Table 6 shows the results of the hierarchical multiple linear regression analyses performed using perceived stress as the dependent variable. The control variables (Time 1 emotional/behavioral problems and gender) entered in the first step accounted for 7% of the variance in adolescents’ perceived stress scale (PSS) during COVID-19, but this step (Model 1) was not significant. Model 2 included the pre-COVID 19 sleep variable. In this model, 37% of the variance in adolescents’ PSS during COVID 19 was accounted for by their pre-COVID-19 reported sleep duration and cognitive-emotional arousal at bedtime (*F*(_2, 63_) = 2.69, *p* < 0.01). Shorter sleep duration and a higher level of cognitive arousal at bedtime were associated with higher levels of PSS during COVID-19, above and beyond that accounted for by the control variables (ΔR^2^ = 18%, *p* < 0.001). Model 3 included the variables that were measured during COVID-19, and showed that poorer sleep quality and a higher level of cognitive-emotional arousal at bedtime during the pandemic were associated with higher PSS during COVID 19, above and beyond the pre-COVID-19 levels of sleep, sleepiness, or arousal at bedtime (ΔR2 = 20%, *p* < 0.001). The fully adjusted model explained 55% of the variance in PSS (*F*(_2, 63_) = 5.20, *p* < 0.01).

**Table 6:** Hierarchical regression of perceived stress during COVID, sleep, arousal at bedtime and daytime sleepiness

	Perceived stress	*B*	*SE B*	*β*	*t*	*R2*	*ΔR2*	*F*
Step 1	Covariate					0.07	0.07	2.28
	Gender	− 2.98	1.78	− 0.21	− 1.67			
	Behavioral/emotional problems	0.11	0.09	0.16	1.26			
Step 2	Time 1					0.25	0.18	2.69*
	Sleep duration	− 1.13	0.57	− 0.26	(− 1.99)*			
	Sleep quality	− 0.09	1.12	− 0.01	− 0.08			
	Daytime sleepiness	0.26	0.21	0.16	1.23			
	Behavioral arousal at bedtime	1.31	0.71	0.25	1.86			
	Cognitive emotional arousal at bedtime	− 1.88	0.94	− 0.31	(− 1.99)*			
Step 3	Time 2							
						0.55	0.30	5.20**
	Sleep duration	− 0.66	0.44	− 0.16	− 1.50			
	Sleep quality	2.40	1.04	0.28	2.32*			
	Daytime sleepiness	− 0.44	0.27	− 0.20	− 1.61			
	Behavioral arousal at bedtime	0.44	0.58	0.09	0.76			
	Cognitive emotional arousal at bedtime	− 4.33	− 4.33	− 0.60	− 4.62			

N, 63; B, unstandardized coefficients; SE B, standard error of unstandardized coefficients; = standardized coefficients

^*^p ≤ .05. **p ≤ 0.01. ***p ≤ 0.001

## Discussion

This longitudinal study prospectively examined the contribution of sleep patterns and behavior before and during the COVID-19 pandemic to adolescents’ perceived stress during the COVID-19 pandemic. To our knowledge, only two other longitudinal studies have prospectively examined changes in adolescents’ sleep following the initiation of the COVID-19 pandemic, and neither one of them prospectively examined the impact of sleep on stress levels.

The study findings indicate that: (1) The adolescents’ sleep duration was longer and the sleep schedule was delayed during the COVID-19 pandemic compared to pre-pandemic sleep. (2) A larger proportion of the adolescents obtained the recommended amount of sleep or more during the COVID-19 pandemic compared to pre-pandemic. (3) “Social jet lag” disappeared during the COVID-19 pandemic. (4) The pre-COVID-19 sleep duration and arousal at bedtime were significant predictors of adolescents’ levels of perceived stress during the COVID-19 pandemic. (5) Higher levels of arousal at bedtime and poorer sleep quality were concurrently associated with higher levels of perceived stress among adolescents during the COVID-19 pandemic, above and beyond their pre-pandemic emotional or behavioral issues, sleep duration, or sleep quality.

The use of objective sleep data to characterize participants’ habitual sleep prior to the COVID-19 pandemic was important for establishing their baseline sleep. The use of both subjective and objective measures of sleep at Time 1 is considered to be a strength of this study, because it allowed us to assess the validity of the participants’ self-reported information regarding sleep. Although self-reported measures can lead to inaccuracies and carry a risk for information bias, the information used in the present study was judged to be acceptable.

Consistent with other reports [35, 40, 59–61], we found that during the COVID-19 pandemic, the bedtime and wake-up time of typically developing adolescents shifted and were delayed by about 2 h, their sleep duration was longer, their daytime sleepiness was markedly reduced, and a significantly larger proportion of them obtained the recommended amount of sleep for their age, compared to the pre-pandemic sleep patterns. In addition, the discrepancy between week and weekend sleep patterns known as “social jet lag” was eliminated.

This could be explained by COVID-19-related societal and psychosocial changes that decreased the discrepancy between adolescents’ delayed biological rhythm and the daily timing determined by social constraints. The delayed school start time allowed adolescents to follow their natural tendency for delayed awakening. Parents might have been more permissive with regards to bedtime during the week, allowing adolescents to follow their natural preference for a delayed bedtime. Remote learning allowed adolescents to sleep longer, as they did not have to commute to school. In addition, the cancelation of extracurricular activities and the need to socially isolate meant that adolescents had more “useable hours” during the weekdays to complete their homework and did not have to sacrifice sleep to fulfill their obligations during the week. The combination of these factors might have prevented the accumulation of sleep debt during the week, thereby reducing or eliminating the need to “catch up on lost sleep” during the weekend.

Collectively, the increased flexibility of sleep timing and decreased cumulative sleep debt during the week caused by multiple environmental and psychoscocial factors might have resulted in the elimination of “social jet lag” during the pandemic.

The findings of the present study demonstrate that changes in societal circumstance that resulted in a delayed school start time and elimination of the morning commute led to an extension of the sleep duration and a significant reduction in adolescents’ daytime sleepiness. Similar findings have been reported in multiple countries around the world during the COVID-19 pandemic, supporting previous scientific findings regarding the impact of a delayed circadian physiology amongst adolescents. These findings indicate that adolescents’ sleep deprivation during the school week and their pattern of “social jet lag” could be prevented by delaying the school start time.

The ability of adolescents to naturally extend their sleep during the pandemic, when school started later, demonstrates that sleep extension is possible in this population, and that a short sleep duration is a modifiable risk factor that could be targeted to protect adolescents from stress and related mental health challenges.

The tendency of adolescents to have a short sleep duration was a global concern prior to the COVID-19 pandemic. Multiple studies found that adolescents were getting less than the recommended amount of sleep and experiencing daytime sleepiness. The findings of the present study show that not only did insufficient sleep during the pre-pandemic period place typically developing adolescents at risk for daytime impairments, it affected their ability to cope in a time of crisis. The adolescents’ sleep duration before the COVID-19 pandemic was a significant predictor of their level of perceived stress during the COVID-19 pandemic, such that shorter sleep duration was associated with higher levels of stress during the pandemic.

The present study extended previous work by using sleep behaviors as independent variables in a prospective study in which perceived stress during the COVID-19 pandemic was the dependent variable. This design revealed that a high pre-pandemic level of arousal at bedtime increased adolescents’ stress’ during the COVID-19 pandemic. Finally, sleep quality and a high level of cognitive-emotional arousal at bedtime were cross-sectionally associated with high levels of stress during the COVID-19 pandemic, above and beyond the impact of these variables at Time 1. In addition, similar to previous reports, we found that cognitive arousal accounted for the variance of perceived stress as compared to somatic arousal.

These findings provide novel insights into the relationship between sleep patterns and behaviors at bedtime with adolescents’ perceived levels of stress, and suggest that sleep extension and reduction of arousal at bedtime should tested as key components of preventative interventions aiming at reducing adolescents’ stress in the face of stressogenic situations.

Several strategies could be used to accomplish this goal. First, delaying adolescents’ school start time could help accomplish this goal and should be implemented by schools interested in supporting the mental health of their students. Second, sleep health should become a standard component of school health curricula. Adolescents should be encouraged to consistently obtain sufficient amount of sleep by being educated on the importance of sleep and given concrete strategies to help them achieve this goal. Third, in light of the key prospective and concurrent associations between high levels of cognitive-emotional arousal at bedtime and high levels of stress, future work should test the impact of integrating into school-based health curricula evidence-based strategies to lower cognitive arousal at bedtime, such as bedtime stimulus control or mindfulness-based stress reduction [62, 63]. Such efforts could have far-reaching impact, given that a higher level of arousal at bedtime among adolescents has been shown to be present prior to the manifestation of insomnia, which increases adolescents’ risk for mood and anxiety disorders.

### Study limitations and future directions

The key strengths of this study include its longitudinal design and use of validated sleep and stress measures. However, several limitations merit consideration. Although our analyses were designed to ascertain specific sleep patterns associated with adolescents’ stress, there may have been fluctuation in stress and sleep that was not captured here. Future longitudinal analyses should examine the extent to which sleep and stress may drive changes in one another.

Another limitation is that this study identified pre-pandemic predictors of high levels of perceived stress, but did not reveal the mechanisms though which they operated. Executive functions are essential for children’s ability to quickly and flexibly adapt to changed circumstances [64, 65]. Previous studies showed that the sleep deprivation in typically developing adolescents is associated with impaired daily executive capacities [66]. Future work should investigate the extent to which executive functions mediate or moderate the impact of a short sleep duration or high level of cognitive-emotional arousal at bedtime on adolescents’ sleep. In addition, future work should seek to delineate the specific aspects of cognition or cognitive processes that should be targeted in cognitive interventions aimed at effectively reducing adolescents’ arousal at bedtime.

### Practical recommendations

This study found that adolescents’ short sleep duration and higher cognitive-emotional arousal at bedtime in the months prior to the COVID-19 pandemic contributed to increased levels of perceived stress during the COVID-19 pandemic. Since these are modifiable behaviors, we recommend that these parameters be assessed and addressed as part of preventive care and in routine clinical practice, as follows:

#### Integration of sleep health promotion into preventive clinical practices

A nurse or physician’s assistant could offer individual or group patient-education sessions intended to promote healthy sleep. In addition, educational videos and brochures could be sent to adolescents electronically and made accessible on the practice website.

The adolescent and their parents could be encouraged to select a school or program that is better suited to the adolescent’s biological clock. If this is not possible, the clinician could help the adolescent and their family select a realistic sleep schedule that would allow the adolescent to obtain a sufficient amount of sleep within the existing school schedule.

#### Integration of sleep hygiene assessment and sleep disorder screening into routine visits and clinical evaluations

We recommended that clinicians ask about the adolescent’s sleep, integrate sleep hygiene assessment into the clinical evaluation, and screen for the presence of pediatric sleep deprivation and disorders. When needed, the clinician should make appropriate referrals for sleep disorders that require specialized care.

#### Integrate healthy sleep practices into your clinical care

When indicated in the assessment, the clinician should encourage adolescents to acquire and use strategies that can help decrease hyperarousal at bedtime, such as relaxation or mindfulness meditation.

## Conclusions

Sleep duration and cognitive-emotional arousal, which are both modifiable behaviors, were associated with adolescents’ perceived stress during the COVID-19 pandemic. These behaviors could be useful targets for preventive interventions aiming to reduce adolescents’ stress in the face of stressogenic situations, such as the COVID-19 pandemic.

## Data Availability

The datasets used and/or analysed during the current study are available from the corresponding author on reasonable request.
